# Early prediction of hospital outcomes in patients tracheostomized for complex mechanical ventilation weaning

**DOI:** 10.1186/s13613-022-01047-z

**Published:** 2022-08-08

**Authors:** Davy Cabrio, Timothée Vesin, Ermes Lupieri, Hélène Messet, Kishore Sandu, Lise Piquilloud

**Affiliations:** 1 Service de Médecine Intensive Adulte, Centre Hospitalier Universitaire Vaudois, BH08/651, Rue du Bugnon 46, 1011 Lausanne, Suisse; 2 Université de Lausanne, Lausanne, Suisse; 3 Service de Médecine Intensive et Réanimation, Centre Hospitalier Régional Universitaire de Tours, Tours, France; 4 Service d’oto-rhino-laryngologie, Centre Hospitalier Universitaire Vaudois, Lausanne, Suisse

**Keywords:** Mechanical ventilation, Prolonged weaning, Outcomes, Tracheostomy, Prediction

## Abstract

**Background:**

Tracheostomy is often performed in the intensive care unit (ICU) when mechanical ventilation (MV) weaning is prolonged to facilitate daily care. Tracheostomized patients require important healthcare resources and have poor long-term prognosis after the ICU. However, data lacks regarding prediction of outcomes at hospital discharge. We looked for patients’ characteristics, ventilation parameters, sedation and analgesia use (pre-tracheostomy) that are associated with favorable and poor outcomes (post-tracheostomy) using univariate and multivariate logistic regressions.

**Results:**

Eighty tracheostomized patients were included (28.8% women, 60 [52–71] years). Twenty-three (28.8%) patients were intubated for neurological reasons. Time from intubation to tracheostomy was 14.7 [10–20] days. Thirty patients (37.5%) had poor outcome (19 patients deceased and 11 still tracheostomized at hospital discharge). All patients discharged with tracheostomy (*n* = 11) were initially intubated for a neurological reason. In univariate logistic regressions, older age and higher body-mass index (BMI) were associated with poor outcome (OR 1.18 [1.07–1.32] and 1.04 [1.01–1.08], *p* < 0.001 and *p* = 0.025). No MV parameters were associated with poor outcome. In the multiple logistic regression model higher BMI and older age were also associated with poor outcome (OR 1.21 [1.09–1.36] and 1.04 [1.00–1.09], *p* < 0.001 and *p* = 0.046).

**Conclusions:**

Hospital mortality of patients tracheostomized because of complex MV weaning was high. Patients intubated for neurological reasons were frequently discharged from the acute care hospital with tracheostomy in place. Both in univariate and multivariate logistic regressions, only BMI and older age were associated with poor outcome after tracheostomy for patients undergoing prolonged MV weaning.

**Supplementary Information:**

The online version contains supplementary material available at 10.1186/s13613-022-01047-z.

## Background

Weaning, the process of liberating the patient from mechanical ventilation, is crucial to improve critically ill patient’s outcome [1]. Tracheostomy in the intensive care unit is a frequent intervention for patients who cannot be weaned from mechanical ventilation (MV) [2]. It was shown in a large multi-center prospective study [1] that 8.7% of patients invasively ventilated have prolonged weaning (defined as the persistent need for MV for 7 days after the first attempt at discontinuing MV) and 4.1% of ventilated patients require tracheostomy.

Among patients with prolonged weaning, we can describe two main groups who need tracheostomy: patients with inadequate airway protection due to neurological impairment and patients with persistent respiratory impairment. In patients suffering from neurological sequelae, tracheostomy helps protect the airway and reduce ventilator-associated pneumonias [3, 4], reduces days with MV [5], facilitates transfer to long-term care facilities [3], but does not reduce mortality [6]. For patients suffering from persistent respiratory impairment, tracheostomy decreases work of breathing [7], sedation needs [8], allows better mobilization and improves patients’ comfort compared to orotracheal intubation [9]. In addition, tracheobronchial toilet is easier [8] and communication with care providers is improved [10]. Once MV is weaned off, oral feeding can often be reintroduced even with tracheostomy cannula still in place [10].

Despite the benefits listed above, tracheostomy can lead to complications, such as tracheal stenosis and stromal bleeding or infections [11]. Tracheostomized patients are also resources demanding. They stay for a long period of time in the ICU, hospital and long-term care facilities. Both patients tracheostomized for non-neurological and neurological problems have high mortality rates of at least 45% at 1 year [12, 13] and poor long-term outcomes [13, 14]. Even the impact of tracheostomy itself on the long-term outcome is poorly known and difficult to individualize from other healthcare and disease-related factors. Poor outcome of tracheostomized patients highlights the importance of further assessing the criteria that could be used to decide which patients are good candidates to benefit from tracheostomy. Recent French guidelines addressed this important question, also underlining that additional data is needed [15]. Only higher body weight [16], presence of comorbidities [17–19] and albuminemia levels [20] have previously been associated with worse outcome in tracheostomized patients. In practice, ICU clinicians use clinical judgment and the general health status of the patient to decide whether to perform tracheostomy. Among unanswered questions, the relationship between ventilator settings, sedation and analgesia administered before tracheostomy and outcome has also not been systematically studied in tracheostomized patients. In addition, no data is available regarding the impact on outcome of performing early and frequent attempts to discontinue MV (spontaneous breathing trial or SBT). Finally, data is also sparse regarding the correlation between MV weaning strategies after tracheostomy and outcome [21, 22]. We hypothesize that patients’ and treatments-related characteristics could help predict outcomes in patients tracheostomized for complex MV weaning.

The main objective of this work was to study, in patients tracheostomized for MV weaning purposes, the association between patients’ outcome at hospital discharge and patients’ characteristics, tracheostomy technique, MV management and sedation and analgesia use before performing the tracheostomy.

## Methods

Retrospective single-center study conducted at the medico-surgical Adult Intensive Care Unit of the Lausanne University Hospital (CHUV), Lausanne, Switzerland. Data were collected from medical files and clinical information system. The present study was approved by the local ethics committee (Commission cantonale d'éthique de la recherche sur l'être humain, protocol number 2019-01403). Due to the nature of collected data, waiver of consent was obtained and only patients who explicitly refused the use of their clinical data for research purposes were excluded. The study was registered on clinicaltrials.org (NCT04987398).

Adult patients admitted to the Adult ICU of the Lausanne University Hospital between May 1st 2017 and November 30th 2018 who were mechanically ventilated for at least 72 h and tracheostomized were considered for inclusion. Exclusion criteria were: patients’ refusal to participate to a research project, tracheostomy performed before ICU admission, tracheostomy performed for ear–nose–throat (ENT) reasons, burns’ victim or pre-existing condition(s) prior to the ICU admission precluding ventilation weaning. For all the included patients weaning from mechanical ventilation and tracheostomy management and weaning were performed following the dedicated procedures available in the Lausanne University Hospital ICU.

Patients’ characteristics at ICU admission and reason for ICU admission were collected. Reason for intubation was also recorded. Clinical frailty score, Nutrition risk screening (NRS) score, Simplified Acute Physiology Score II (SAPS II) and Sequential Organ Failure Assessment (SOFA) score were collected as well. Key dates during hospital stay (admission and discharge from ICU and hospital, intubation day, tracheostomy day and definitive cannula ablation day) were collected. Ventilator settings were collected once daily at 8 a.m. except the day of tracheostomy. Ventilatory mode used for the majority of time during each day was collected between intubation and the day before tracheostomy. The use or not of sedation, analgesia and neuromuscular blocking agents (NMBA) was collected every day between intubation and the day before tracheostomy. Dynamic plateau pressure was measured by the ventilator in volume-assist control (VAC), during a short tele-inspiratory pause set by default for each breath (set at 10–15% of the total inspiratory time). Driving pressure was calculated as the difference between dynamic plateau pressure and set PEEP. Data about medication are reported as the percentage of days with use of each medication before tracheostomy. Separation attempts from MV before tracheostomy were considered as either spontaneous breathing trials (SBT) or immediate extubation without previous SBT. They were recorded until the day before tracheostomy. On the day of tracheostomy, ventilation mode and settings were collected every 30 min during the 2 h before intervention and were averaged. Maximal norepinephrine infusion rate administered during those 2 h was collected. SOFA score was also calculated and the worst PaO_2_/FiO_2_ ratio on the day of tracheostomy was recorded. Tracheostomy technique (surgical or percutaneous) and the type of cannula inserted were collected. As general hospital stay data, we collected ICU and hospital mortality, unexpected death vs death following withdrawal of life-sustaining treatments (WLST), ICU and hospital stay durations, days free from MV at days 30 and 60 after intubation, decannulation, time from intubation to decannulation and presence of ICU-acquired weakness when reported in the ICU discharge letters and defined either by a Medical Research Council (MRC) sum score of less than 48/60, a compatible electroneuromyography exam or high clinical suspicion in the absence of sufficient collaboration to perform MRC scale. More details on data collection are available in Additional file 1. Missing data were not imputed.

Favorable outcome was considered when the patient was alive and decannulated at hospital discharge. Contrarily, poor outcome was considered as in-hospital death or discharge with tracheostomy cannula in place. Patients were divided into two sub-groups depending on their outcome (favorable vs poor).

No statistical sample size calculation was performed a priori for this retrospective study. Sample size was equal to the number of patients treated during the study period who met inclusion criteria and did not meet exclusion criteria.

### Data analyses

Data was reported as median [interquartile range] or number (percentage). Normality was tested using Shapiro–Wilk test. Comparisons between outcome groups for continuous data were performed using *T* test or Mann–Whitney test as appropriate. Fisher’s exact test was used for categorical data. Binary logistic regressions were used to evaluate the association of pre-tracheostomy variables and of tracheostomy technique with patients’ outcome. These analyses were performed for both the global patients’ population and the subgroup of patients intubated for non-neurological reasons. Respiratory rate was not included in the univariate analyses, because it represents both a ventilator setting (controlled ventilation) and the patient’s own respiratory rate if present (assisted ventilation). A multivariate logistic regression model was constructed both for the global patients’ population and for patients intubated for non-neurological reasons to identify variables independently associated with favorable or poor outcomes. Variable entered in the multivariate model were those with univariate *p* value of < 0.10. Results for univariate and multivariate logistic regression models were reported as odds ratio (OR) and 95% confidence interval (CI). Parameters significantly associated with outcomes in the multivariate regressions model were compared between the patients intubated for neurological, respiratory and other reasons using ANOVA or Kruskal–Wallis test as appropriate. Statistical analyses were performed using GraphPad Prism version 9.1.0 for Windows (GraphPad Software, San Diego, CA, USA) except for Fisher’s exact tests, which were performed using *R* version 1.4.2 (R Foundation for Statistical Computing, Vienna, Austria). All statistical tests were two-tailed and *p* value < 0.05 was considered significant.

## Results

### Study population

**Fig. 1:**
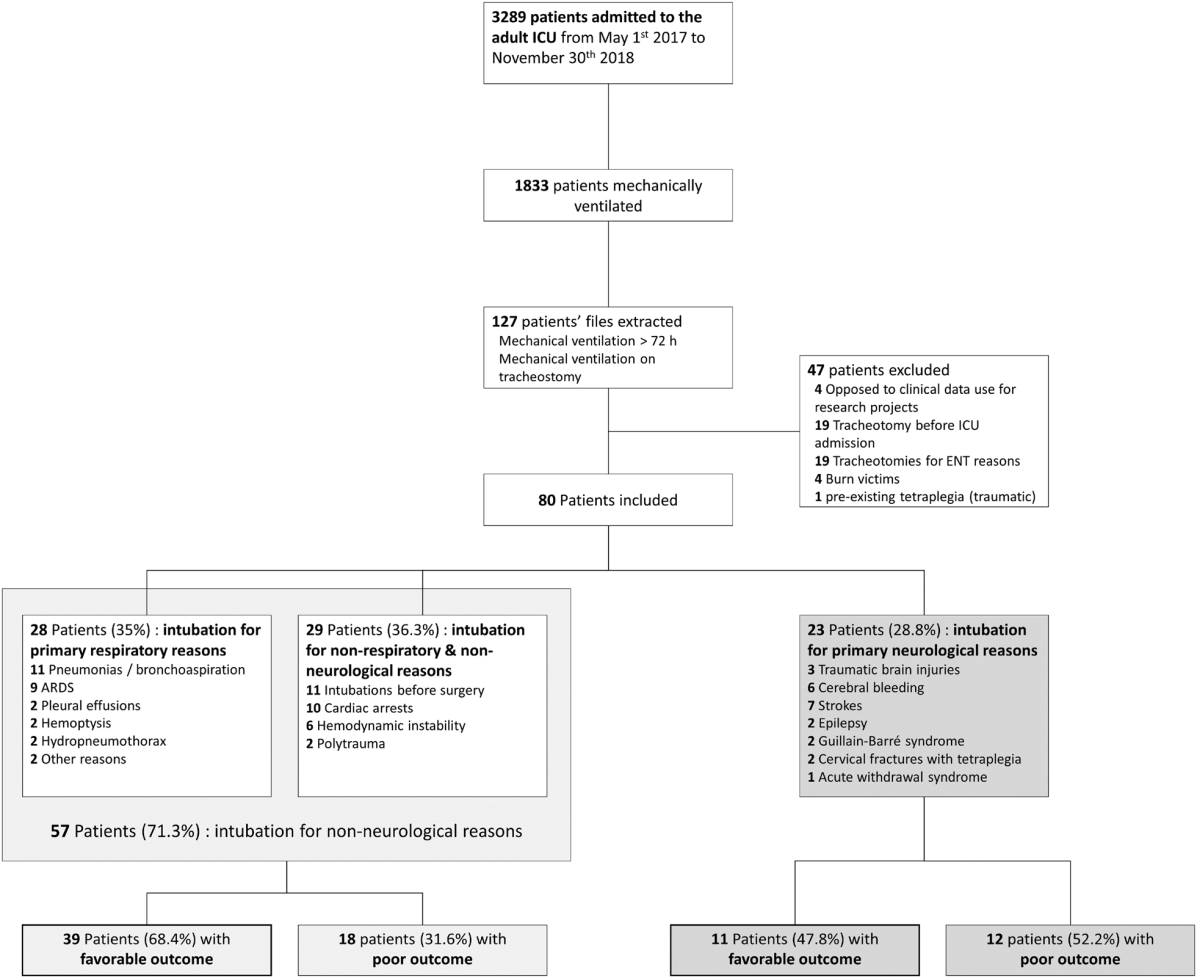
Study flowchart. *ICU* intensive care unit, *ENT* ear–nose–throat, *ARDS* acute respiratory distress syndrome

A total of 80 patients were included. Twenty-three patients were intubated for neurological reasons. Twenty-eight were intubated for primary respiratory reasons and 29 for non-neurological and non-respiratory reasons. Those two last sub-groups had similar characteristics (see Additional file 2) and were analyzed as a single sub-group (*N* = 57). The study flowchart is displayed in Fig. 1. No complications related to the insertion procedure of tracheostomy (performed by an ENT specialist or a thoracic/abdominal surgeon) were observed. All the cannula used were Shiley (Covidien, Minneapolis MN, USA), size 6–10.

### Patients’ characteristics and outcomes

Patients’ characteristics, ICU admission data and reasons leading to intubation are included in Table 1 for the global study population and after separation in favorable and poor outcome. General characteristics and some comorbidities data are provided for both sub-groups of patients intubated for non-neurological and neurological reasons in Additional file 3 and Additional file 4, respectively.

**Table 1:** Patients’ general characteristics, comorbidities and admission data

	Study population	Favourable outcome	Poor outcome	
	*N* = 80*	*N* = 50*	*N* = 30*	*p value*^*#*^
General characteristics				
Age, year	60 [52–71]	59 [50–67]	68.5 [55–76]	*0.03*
Women, *n* (%)	23 (28.8%)	16 (32%)	7 (23.3%)	
BMI, kg/m^2^	25.6 [21–30]	24.2 [21–27]	28.2 [24–32]	< *0.01*
Comorbidities				
Pulmonary comorbidities				
Obstructive disease, *n*. (%)	18 (22.5%)	10 (20%)	8 (26.7%)	*0.58*
Restrictive disease, *n*. (%)	1 (1.3%)	1 (2%)	0 (0%)	*1*
OAS, *n*. (%)	9 (11.3%)	5 (10%)	4 (13.3%)	*0.72*
Other pulmonary disease, *n*. (%)	2 (2.5%)	2 (4%)	0 (0%)	*0.53*
Home O_2_-therapy, *n*. (%)	2 (2.5%)	2 (4%)	0 (0%)	*0.53*
Home NIV-therapy, *n*. (%)	3 (3.8%)	2 (4%)	1 (3.3%)	*1*
Cardiac comorbidities				
Coronary artery disease, *n*. (%)	6 (7.5%)	5 (10%)	1 (3.3%)	*0.40*
Heart failure, *n*. (%)	0 (0%)	0 (0%)	0 (0%)	*1*
Other comorbidities				
Chronic kidney disease, *n*. (%)	4 (5%)	1 (2%)	3 (10%)	*0.15*
Active neoplasia, *n*. (%)	22 (27.5%)	15 (30%)	7 (23.3%)	*0.61*
Central neurological disease, *n*. (%)	1 (1.3%)	1 (2%)	0 (0%)	*1*
Clinical Frailty Score	3 [2–5]	3 [2–5]	4 [2–5]	*0.83*
NRS score at admission	6 [3–6]	6 [3–6]	5.5 [4–6]	*0.37*
Admission data				
Reason for ICU admission				*0.31*
Cardiac arrest	5 (6.3%)	3 (6%)	2 (6.7%)	
Oliguria/anuria/CRRT need	2 (2.5%)	1 (2%)	1 (3.3%)	
Respiratory distress	20 (25%)	15 (30%)	5 (16.7%)	
Shock	9 (11.3%)	5 (10%)	4 (13.3%)	
Post-operative (planned)	6 (7.5%)	6 (12%)	0 (0%)	
Post-operative (emergency surgery)	11 (13.8%)	4 (8%)	7 (23.3%)	
Polytrauma	6 (7.5%)	4 (8%)	2 (6. 7%)	
Other hospital transfer	5 (6.3%)	3 (6%)	2 (6. 7%)	
Altered level of consciousness	16 (20%)	9 (18%)	7 (23.3%)	
Type of ICU admission				*1*
Medical, *n*. (%)	28 (35%)	18 (36%)	10 (33.3%)	
Surgical, *n*. (%)	52 (65%)	32 (64%)	20 (66. 7%)	
SAPS II at admission	46.5 [39–62]	44.5 [36–64]	51.0 [43–61]	*0.33*
SOFA Score at admission	9.0 [7–11]	8.0 [7–11]	9.0 [7–11]	*0.89*
Neurological reason for intubation	23 (28.8%)	11 (22%)	12 (40%)	0.13

*N = 80, except for NRS score at admission, where *N* = 57 (*N* = 35 for favourable outcome, *N* = 22 for poor outcome)

*BMI* body mass index, *OAS* obstructive apnea syndrome, *NIV* non-invasive ventilation, *NRS* nutrition risk screening, *ICU* intensive care unit, *CRRT* continuous renal replacement therapy, *SAPS II* Simplified Acute Physiology Score II, *SOFA score* Sequential Organ Failure Assessment score

^#^*p* value calculated using *t* test or Mann–Whitney test for continuous data and Fisher’s exact test for categorical data

In the global study population, 19 (23.8%) patients died during hospital stay, 9 in the ICU (ICU mortality of 11.3%) and 10 after ICU stay. One patient died of direct complication of tracheostomy-related adverse event (accidental decannulation). Among all deceased patients, 12 (63.2% of all deceased patients) died after WLST. Seven WLST were conducted in the ICU and 5 after the ICU stay. To note, no patients in the favourable outcome group had WLST during the hospital stay. For 10/61 (16.4%) patients alive at hospital discharge, a “do not resuscitate order in case of cardiac arrest” was found in the medical record.

In patients intubated for neurological reasons, 3 out of 23 (13%) died during hospital stay, one in the ICU (WLST) and 2 after ICU stay (non-WLST). In patients intubated for non-neurological reasons, 16 out of 57 (28.1%) died during hospital stay, 8 in the ICU (6 WLST and 2 non-WLST) and 8 after ICU stay (5 WLST and 3 non-WLST). Hospital mortality tended to be lower in patients intubated for neurological reasons than for non-neurological reasons (13% vs 28.1%, *p* = *0.245*) but the difference was not significant.

Among the global study population, 30 patients (37.5%), were classified as poor outcome, 19 because of death and 11, because tracheostomy cannula was not weaned during acute care hospital stay. Those 11 patients were all intubated for neurological reasons. General hospital data are displayed in Table 2 for the global population and in Additional file 3 and Additional file 4 both sub-groups.

**Table 2:** General hospital data

		Study population		Favourable outcome		Poor outcome	
	*N*		*N*		*N*		*p *value
ICU stay duration, days	80	29.5 [20–44]	50	28.5 [21–45]	30	29.5 [20–43]	*0.82*
Tertiary hospital stay duration, days	80	55 [43–78]	50	57 [46–90]	30	49 [37–64]	*0.02*
Days free of MV at day 30, days	72	3.7 [0–12]	47	5 [0–12]	25	0.8 [0–12]	*0.71*
Days free of MV at day 60, days	72	32.9 [20–41]	47	35 [23–42]	25	29.9 [1–37]	*0.11*
Intubation to cannula ablation during or after acute care hospital stay, days	54	42 [35–58]	49	40 [34–48]	5	76 [61–144]	< *0.01*
ICU-acquired weakness diagnosis, *n*. (%)	80	20 (25%)	50	13 (26%)	30	7 (23. 3%)	*0.79*
With MRC score < 48/60, *n*. (%)	80	17 (21.3%)	50	11 (22%)	30	6 (20%)	*1*
With EMNG/high clinical suspicion, *n*. (%)	80	3 (3.8%)	50	2 (4%)	30	1 (3.3%)	*1*
MRC score value	17	20 [0.5–32.5]	11	20 [0–33]	6	17.5 [5.3–35.5]	*0.9*

*ICU* intensive care unit, *MV* mechanical ventilation, *MRC* medical research council sum score, *EMG* electromyography

^#^*p* value calculated using *T* test or Mann–Whitney test for continuous data and Fisher’s exact test for categorical data

### Data from intubation to tracheostomy

Main ventilator settings and monitored parameters, separation attempts, use of sedation, opioids and NMBA for the period from intubation to the day before tracheostomy are mentioned in Table 3 for the global population and for patients with favorable and poor outcomes. The same information is mentioned for the subgroups of patients intubated for non-neurological or neurological reasons in Additional file 3 and Additional file 4. SOFA score on the day of tracheostomy, the worst PaO_2_/FiO_2_ ratio on the day of tracheostomy, tracheostomy technique and time from intubation to tracheostomy are presented in Table 3 for the global population and in Additional file 3 and Additional file 4 for the subgroups of patients intubated for non-neurological and neurological reasons.

**Table 3:** Ventilation data, sedation, opioids, NMBA use and tracheostomy data

	Study population	Favourable outcome	Poor outcome	
	*N* = 80*	*N* = 50*	*N* = 30*	*p value#*
Ventilation data between intubation and tracheostomy				
Percentage of mechanical ventilation days with more than 12 h with:				
VAC, *n*. (%)	33.3% [18–59%]	39.3% [22–60%]	28.7% [10–48%]	*0.16*
PAC, *n*. (%)	0% [0–0%]	0% [0–0%]	0% [0–0%]	*0.85*
PSV, *n*. (%)	61.1% [40–79%]	57.7% [40–74%]	66.7% [46–86%]	*0.22*
Other, *n*. (%)	0% [0–0%]	0% [0–0%]	0% [0–0%]	*0.55*
V_T_, mL	454.6 [414–530]	441.2 [403–532]	469.5 [424–533]	*0.29*
V_T_/PBW, mL/kg	7 [6–8]	7 [6–8]	7 [6–8]	*0.57*
PEEP, cmH_2_O	6.9 [6–8]	6.8 [6–8]	7.1 [6–8]	*0.10*
RR, cycle/min	21.8 [20–25]	22.6 [20–25]	21.5 [18–26]	*0.94*
Dynamic P_plat_, cmH_2_O	20.3 [18–24]	21.7 [18–25]	20 [17–22]	*0.10*
Driving pressure, cmH_2_O	13.4 [11–15]	13.9 [12–17]	12.2 [10–14]	*0.04*
Separation attempts				*0.57*
* 0*	21 (26.3%)	11 (22%)	10 (33.3%)	
* 1*	11 (13.8%)	8 (16%)	3 (10%)	
* 2*	11 (13.8%)	6 (12%)	5 (16.7%)	
> *2*	37 (46.3%)	25 (50%)	12 (40%)	
Percentage of days with sedation use				
Any sedation, %	93.5% [76–100%]	100% [79–100%]	89.3% [66–100%]	*0.09*
Propofol, %	74.3% [50–91%]	77.8% [55–95%]	69% [49–86%]	*0.29*
Midazolam, %	19.1% [0–53%]	33.3% [9–54%]	12.9% [0–34%]	*0.06*
Dexmedetomidine, %	0% [0–21%]	9.8% [0–25%]	0% [0–13%]	*0.11*
Percentage of days with opioids use				
Opioids, %	100% [87–100%]	100% [89–100%]	100% [77–100%]	*0.77*
Morphine, %	0% [0–0%]	0% [0–0%]	0% [0–2%]	*0.30*
Fentanyl, %	94.2% [67–100%]	93.8% [72–100%]	96.1% [53–100%]	*0.88*
Other opioids, %	0% [0–0%]	0% [0–0%]	0% [0–0%]	*0.80*
Percentage of days with NMBA use				
NMBA, %	12.5% [0–30%]	14.4% [4–31%]	10.4% [0–28%]	*0.40*
Proportion of patients receiving sedation or opioids the day before tracheostomy				
Sedation, *n* (%)	60 (75.0%)	38 (76%)	22 (73.3%)	*0.80*
Opioids *n* (%)	67 (83.75%)	43 (86%)	24 (80%)	*0.54*
Tracheostomy data				
Worst PaO_2_/FiO_2_ ratio on the day of tracheostomy				*0.50*
≥ 400 mmHg	2 (2.5%)	1 (2%)	1 (3.3%)	
< 400 mmHg	12 (15%)	5 (10%)	7 (23.3%)	
< 300 mmHg	18 (22.5%)	13 (26%)	5 (16.7%)	
< 200 mmHg	43 (53.8%)	28 (56%)	15 (50%)	
< 100 mmHg	5 (6.3%)	3 (6%)	2 (6.7%)	
Type of tracheostomy				*0.76*
Percutaneous, *n* (%)	13 (16.25%)	9 (18%)	4 (13. 3%)	
Surgical, *n* (%)	67 (83.75%)	41 (82%)	26 (86. 7%)	
Time from intubation to tracheostomy, days	14.7 [10–20]	14.6 [10–20]	14.8 [10–22]	*0.92*

*N* = 80 except for dynamic *P*_plat_ and driving pressure, where *N* = 70 (*N* = 45 for favourable outcome and N = 25 for poor outcome)

*VAC* volume assist-control, *PSV* pressure-support ventilation, *PAC* pressure assist-control ventilation, *VT* tidal volume, *PBW* predicted body-weight, *PEEP* positive end-expiratory pressure, *RR* respiratory rate, *P*_plat_ plateau pressure, *NMBA* neuromuscular blocking agents, V_T_, V_T_/PBW, PEEP, RR and Dynamic P_plat_ were recorded once a day at 8 am

^#^*p* value calculated using *T* test or Mann–Whitney test for continuous data and Fisher’s exact test for categorical data

Use of sedation and opioids the day before tracheostomy, ventilation data and norepinephrine infusion rate 2 h before tracheostomy are mentioned in Additional file 5.

**Fig. 2:**
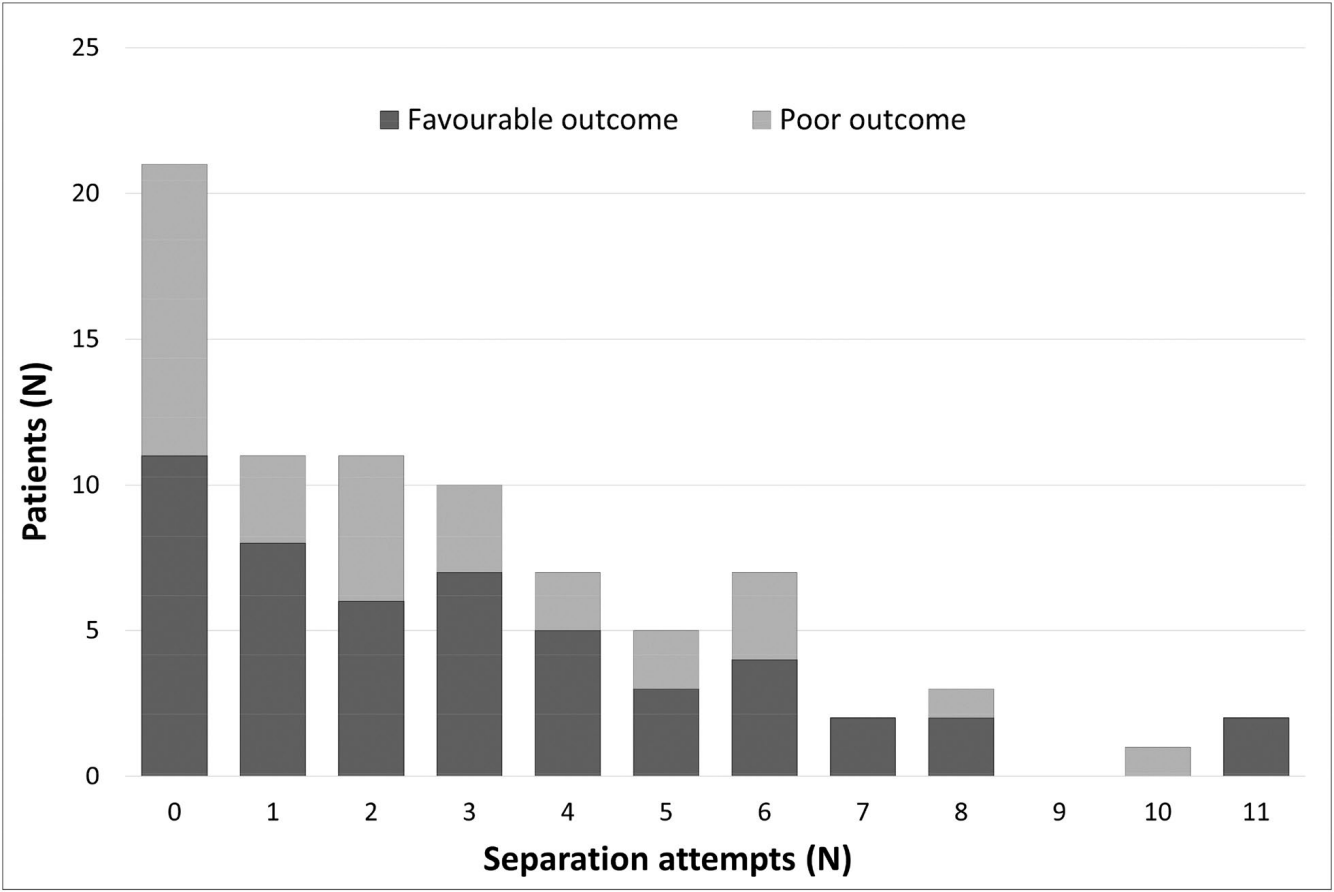
Separation attempts before tracheostomy. Number of separation attempts by patients for each group of outcome

Figure 2 illustrates the number of patients with favorable and poor outcome according to the number of separation attempts before tracheostomy.

### Factors associated with outcome

Table 4 summarizes for the global population the results of univariate logistic regressions and multivariate analysis. In univariate logistic regressions, older age and higher BMI were associated with poor outcome, with OR of 1.18 [1.07–1.32] and 1.04 [1.01–1.08] (*p* < *0.001* and *0.025*, respectively). As post-hoc analysis, a second multivariate model with duration from intubation to tracheostomy forced into the model because of its clinical relevance was performed. This model did not show different results (see Additional file 6). We also conducted univariate logistic regressions and multivariate analysis for the sub-group of patients intubated for non-neurological reasons. The univariate analyses revealed only age as a factor associated with poor outcome (OR of 1.054 [1.01–1.11] *(p* = *0.0191)*). The multivariate model showed that BMI and age were associated with poor outcome in this sub-group of patients intubated for non-neurological reasons. Detailed results of the univariate and multivariate analyses are mentioned in Additional file 7.

**Table 4:** Univariate analyses and multivariate logistic regression model for factors potentially associated with bad outcome

	Univariate regression		Multivariate model		
	OR (CI 95%)	*p *value	OR (CI 95%)	VIF	*p *value
BMI	1.181 (1.07–1.32)	0.0009	1.205 (1.09–1.36)	1.003	0.0008
Age	1.038 (1.01–1.08)	0.0253	1.044 (1.00–1.09)	1.037	0.0463
Sex	2.061 (0.73–6.42)	0.2967			
Number of comorbidities	1.248 (0.73–2.15)	0.4471			
Clinical Frailty Score	0.986 (0.76–1.27)	0.8269			
NRS score at ICU admission	1.129 (0.88–1.48)	0.385			
SAPS II at ICU admission	1.011 (0.99–1.04)	0.3227			
SOFA score at ICU admission	1.010 (0.87–1.17)	0.8776			
Type of ICU admission (medical/surgical)	0.560 (0.22–1.44)	0.3203			
Neurological cause for intubation	0.849 (0.30–2.30)	0.8038			
V_T_/PBW	1.160 (0.81–1.68)	0.5643			
PEEP	1.026 (0.79–1.34)	0.996			
Dynamic plateau pressure	0.898 (0.78–1.02)	0.1097			
Percentage of days with sedation use	0.213 (0.03–1.26)	0.1011	0.208 (0.02–1.57)	1.035	0.14
Percentage of days with opioids use	0.293 (0.02–5.01)	0.7923			
Percentage of days with NMBA use	0.592 (0.07–4.19)	0.401			
Control ventilation before tracheostomy	0.383 (0.07–1.97)	0.1597			
1st separation attempt	1.062 (0.95–1.19)	0.2298			
Any separation attempt	0.564 (0.20–1.57)	0.3983			
Sedation use (day before tracheostomy)	1.556 (0.54–4.91)	0.551			
Opioids use (day before tracheostomy)	3.949 (0.96–26.82)	0.2531			
Tracheostomy technique (percutaneous vs surgical)	1.427 (0.42–5.70)	0.728			
Time from intubation to tracheostomy	1.006 (0.95–1.07)	0.921			

*BMI* body mass index, *NRS* nutrition risk screening, *ICU* intensive care unit, *SAPS II* Simplified Acute Physiology Score II, *SOFA score* Sequential Organ Failure Assessment score, *V*_*T*_*/PBW* tidal volume divided by predicted body weight, *PEEP* positive end-expiratory pressure, *NMBA* neuromuscular blocking agents

Left *p* values calculated using univariate logistic regression for each variable. Right *p* values calculated with multiple logistic regression model, which included BMI, age and sedation use

## Discussion

We reviewed ventilation settings, sedation–analgesia and outcomes of patients ventilated for more than 72 h and tracheostomized during the ICU stay, both for neurological and non-neurological reasons. For the global patient group, in univariate logistic regressions, only older age and higher BMI were associated with poor outcome, defined as in-hospital death or hospital discharge without decannulation. This remained true in the multivariate logistic regression analysis. For the subgroup of patients intubated for non-neurological reasons, the multivariate analysis led to similar conclusions. In this study, we also confirmed high ICU-admission severity scores, high hospital mortality and long ICU and hospital length of stay in tracheostomized patients [1].

Patients intubated and ventilated for all causes and tracheostomized for difficult weaning have high mortality [14, 23]. For example, in the population of patients ventilated for more than 10 days and tracheostomized following acute respiratory distress syndrome, high 28-day and 90-day mortality was reported (30.8% and 45.2%, respectively [12]). Our study population has the characteristics of a general ICU population, including both medical and surgical patients and patients intubated both for neurological and non-neurological reasons. We found relatively low ICU mortality for tracheostomized patients initially intubated for neurological reasons compared to other studies [24, 25] but a high hospital mortality in line with the literature for the global group of patients [1]. The high hospital mortality observed in our population was expected, considering the high severity scores at admission. Death after withdrawal of life-sustaining therapy concerned 63.2% of patients, suggesting frequent poor evolution after tracheostomy, underlining the difficulty of predicting global evolution at the time of tracheostomy.

In the literature, ICU and hospital length of stay differ in tracheostomized patients depending on the series of patients. Hospital length of stay in our population was higher compared to most available data. This could be related to differences in health care policies. Indeed, long-term weaning facilities are not available in Switzerland. In addition, most long-term care facilities do not manage mechanical ventilation in tracheostomized patients and home discharges with home ventilation on tracheostomy is unusual in Switzerland. Those factors could explain the prolonged length of stay in acute-settings hospital. Local practices regarding late or early withdrawal of life-sustaining treatment could also impact hospital length of stay and this can differ between countries, hospitals and even health-care practitioners [26]. Our high percentage of WLST combined with high length of hospital stay, when compared to other countries in Europe and in the world [27] underline the fact that WLST only takes place relatively late in our hospital in tracheostomized patients. This emphasizes the fact that predicting outcomes of tracheostomized patients takes time and requires, at least in Switzerland, a multi-disciplinary consensus, which is sometimes difficult to reach. Such a consensus can sometimes even be difficult to find within the team in charge of the patient because of different individual perceptions of the clinical situation. This is true not only at time of tracheostomy, but also after ICU discharge.

In addition to common ICU parameters to predict outcomes, our study specifically assessed the relationship between outcome and ventilation, sedation, opioid and NMBA use before tracheostomy. No ventilation parameters showed any association with poor outcome. To note, our data also showed good compliance with international ventilation guidelines [28]. Sedation, opioids and NMBA use before tracheostomy did not show any association with outcome. This remained true for tracheostomized patients who intubated for neurological and non-neurological reasons.

This study adds a new perspective on the prediction of unfavorable outcome in tracheostomized patients by demonstrating that ventilation data prior to the tracheostomy did not help predict outcome. Indeed, only higher BMI and older age were associated with poor outcome. This highlights yet the fact that decision to undergo tracheostomy can only be based on general clinical judgment and that more or less severe respiratory status and worse ventilation parameters cannot be used to select patients who could benefit from tracheostomy.

Separation attempts were performed in 73.4% of patients before tracheostomy, which is similar to numbers reported in the recent WIND study collective [1]. The number of separation attempts before tracheostomy was not associated with better or worse outcome. We initially hypothesized that the relationship between mortality and the number of SBT takes the form of a U-shaped curve, with worse outcomes in patients with no SBT (i.e., because of persistent organ failure) and in patients with many SBT (i.e., very prolonged weaning). Therefore, we looked in our data but found no pattern to corroborate this hypothesis. To note, this study did not address the subject of MV weaning strategies after tracheostomy.

As limitations for this study, we must mention that, in this retrospective study, mechanical ventilation data were collected at arbitrary time-points and do not always accurately represent 24-h data. However, as data were collected daily, the ventilation parameters reflect the whole duration of mechanical ventilation before tracheostomy. Second, regarding patients sub-groups, it can be argued that patients intubated because of cardiac arrest could have been classified as patients with neurological impairment. However, eight of them were tracheostomized because of difficult weaning and one because of difficult secretion management. Only one patient was tracheostomized because of persistent neurological impairment. Thirdly, no comparison with a control group without tracheostomy was performed. Even if this comparison would be interesting to assess the impact of tracheostomy on patients’ outcome, we could not do it. Tracheostomy is part of the protocolized management of prolonged weaning in our ICU in the absence of poor prognosis regarding recovery potential. Patients with prolonged weaning who are not tracheostomized have thus different characteristics compared to the tracheostomized patients and cannot be used as a control group. Fourthly, because of the monocentric nature of this study and very different practices regarding tracheostomy between different centers due to the lack of unifying guidelines for tracheostomy indications, our conclusions can probably not be generalized to all other ICUs. Health-care policies and organizational differences concerning the transfer from the ICU to a step-down unit (or other health-care facilities) can also limit the applicability of our results to other hospitals. Finally, in the absence of sample size calculation, our study could potentially have been underpowered to evaluate the association between some factors and outcome. However, tracheostomies for prolonged weaning in the ICU is relatively rare, and monocentric studies rarely have much larger collectives.

## Conclusions

This study showed high mortality and long duration of hospital stay in a medico-surgical population of patients tracheostomized in the ICU in part of the process of MV weaning. In univariate logistic regressions, older age and higher BMI were associated with poor outcome, defined as in-hospital death or hospital discharge without decannulation. This remained true in the multivariate logistic regression analysis. The same factors associated with outcome were identified when the multivariate analysis was performed in the subgroup of patients intubated for non-neurological reasons. We found no association between ventilatory data before tracheostomy and outcome, neither for the global patient population nor for the patients intubated for non-neurological reasons. This was also true for sedation, analgesics and NMBA use up to the day before tracheostomy. Separation attempts were frequent before tracheostomy but the number of attempts was not associated with outcome.

## Supplementary Information

**Additional file 1.**
 Data collection details.

**Additional file 2.**
 Patients’ general characteristics, comorbidities and admission data for study population andseparated by cause for intubation.

**Additional file 3.**
 General characteristics & comorbidities, admission data, ventilation data, sedation, opioids, NMBA use, tracheostomy data and outcomes data for patients intubated for non-neurological reasons.

**Additional file 4.**
 General characteristics & comorbidities, admission data, ventilation data, sedation, opioids, NMBA use, tracheostomy data and outcomes data with patients intubated for neurological reasons only.

**Additional file 5.**
 Ventilation and norepinephrine use 2-hours before tracheostomy.

**Additional file 6.**
 Univariate and multivariate logistic regression models for factors potentially associated with poor outcome.

**Additional file 7.**
 Univariate analyses and multivariate logistic regression model for factors potentially associated with poor outcome for patients intubated for non-neurological reasons only.

## Data Availability

The data sets used and/or analysed during the current study are available from the corresponding author on reasonable request**.**

## Abbreviations

ICU: Intensive care unit
MV: Mechanical ventilation
ENT: Ear–nose–throat
BMI: Body-mass index
CHUV: Centre Hospitalier Universitaire Vaudois
NRS: Nutrition Risk Screening
SAPS II: Simplified Acute Physiology Score II
SOFA: Sequential Organ Failure Assessment
NMBA: Neuromuscular blocking agent
SBT: Spontaneous breathing trial
WLST: Withdrawal of life-sustaining treatment
OR: Odds ratio
CI: Confidence interval
ESM: Electronic supplemental materials

## Declarations

### Notation of prior abstract publication/presentation

This study was presented as an abstract and poster at the Swiss Society of Intensive Medicine (SSMI) on September 22nd 2021 (Interlaken, Switzerland).
