# Supplementary material for: Early prediction of hospital outcomes in patients tracheostomized for complex mechanical ventilation weaning
Source: Ann Intensive Care. 2022 Aug 8;12:73. doi: 10.1186/s13613-022-01047-z (PMC9357593; doi:10.1186/s13613-022-01047-z)
Supplement: Supplementary file 1 — Additional file 1. Data collection details. [file 13613_2022_1047_MOESM1_ESM.docx]

# Additional file 2

Patients’ general characteristics, comorbidities and admission data for study population and separated by cause for intubation.

|  | **Study population** | **Respiratory cause of intubation** | **Neurological cause of intubation** | **Non-neurological & non-respiratory cause of intubation** | *p-value^#^ (global)* |
| --- | --- | --- | --- | --- | --- |
|  | N = 80 | N = 28 | N = 23 | N = 29 |  |
| **General characteristics** |  |  |  |  |  |
| Age - yr | 60 [52 - 71] | 63.5 [51 - 75] | 59 [48 - 70] | 59 [53 - 69] | *ns* |
| Women – n. (%) | 23 (28.8%) | 10 (35.7%) | 8 (34.8%) | 5 (17.2%) | *ns* |
| BMI - kg/m^2^ | 25.6 [21 - 30] | 23.5 [20 - 27]^b^ | 26.4 [24 - 31]^a^ | 26.1 [22 - 30] | *0.024* |
| **Comorbidities** |  |  |  |  |  |
| Pulmonary comorbidities |  |  |  |  |  |
| *Obstructive disease – n. (%)* | 17 (21.3%) | 7 (25%) | 2 (8.7%) | 8 (27.6%) | *ns* |
| *Restrictive disease – n. (%)* | 1 (1.3%) | 1 (3.6%) | 0 (0%) | 0 (0%) | *ns* |
| *OAS – n. (%)* | 8 (10%) | 2 (7.1%) | 1 (4.3%) | 5 (17.2%) | *ns* |
| *Other pulmonary disease – n. (%)* | 6 (7.5%) | 5 (17.9%) | 0 (0%) | 1 (3.4%) | *0.036* |
| Cardiac comorbidities |  |  |  |  |  |
| *Coronary artery disease – n. (%)* | 6 (7.5%) | 2 (7.1%) | 0 (0%) | 4 (13.8%) | *ns* |
| *Heart failure – n. (%)* | 14 (17.5%) | 6 (21.4%) | 1 (4.3%) | 7 (24.1%) | *ns* |
| Other comorbidities |  |  |  |  |  |
| *Chronic kidney disease – n. (%)* | 4 (5%) | 2 (7.1%) | 0 (0%) | 2 (6.9%) | *ns* |
| *Active neoplasia – n. (%)* | 22 (27.5%) | 11 (39.3%) | 3 (13%) | 8 (27.6%) | *ns* |
| *Central neurological disease – n. (%)* | 1 (1.3%) | 1 (3.6%) | 0 (0%) | 0 (0%) | *ns* |
| Clinical Frailty Score | 3 [2 - 5] | 4.5 [3 - 6]^b^ | 2 [2 - 3]^a,c^ | 4 [3 - 5]^b^ | *< 0.001* |
| NRS score at admission | 6 [3 - 6] | 6 [6 - 7] | 4 [3 - 6] | 4 [3 - 6] | *0.006* |
| **Admission data** |  |  |  |  |  |
| Reason for ICU admission |  | ^b,c^ | ^a,c^ | ^a,b^ | *< 0.001* |
| *Cardiac arrest* | 5 (6.3%) | 0 (0%) | 0 (0%) | 5 (17.2%) |  |
| *Oliguria / anuria / CRRT need* | 2 (2.5%) | 1 (3.6%) | 0 (0%) | 1 (3.4%) |  |
| *Respiratory distress* | 20 (25%) | 17 (60.7%) | 0 (0%) | 3 (10.3%) |  |
| *Shock* | 9 (11.3%) | 2 (7.1%) | 0 (0%) | 7 (24.1%) |  |
| *Post-operative (planned)* | 6 (7.5%) | 1 (3.6%) | 0 (0%) | 5 (17.2%) |  |
| *Post-operative (emergency surgery)* | 11 (13.8%) | 1 (3.6%) | 6 (26.1%) | 4 (13.8%) |  |
| *Polytrauma* | 6 (7.5%) | 0 (0.0%) | 4 (17.4%) | 2 (6.9%) |  |
| *Other hospital transfer* | 5 (6.3%) | 2 (7.1%) | 2 (8.7%) | 1 (3.4%) |  |
| *Altered level of consciousness* | 16 (20%) | 4 (14.3%) | 11 (47.8%) | 1 (3.4%) |  |
| Type of ICU admission |  |  |  |  | *ns* |
| *Medical – n. (%)* | 28 (35%) | 12 (42.9%) | 6 (26.1%) | 10 (34.5%) |  |
| *Surgical – n. (%)* | 52 (65%) | 16 (57.1%) | 17 (73.9%) | 19 (65.5%) |  |
| SAPS II at admission | 46.5 [39 - 62] | 43.5 [40 - 59] | 46 [41 - 60] | 58 [39 - 67] | *ns* |
| SOFA Score at admission | 9 [7 - 11] | 10 [8 - 12] | 8 [6 - 10]^c^ | 9 [6 - 11]^b^ | *0.014* |

** N = 80, except for NRS score at admission where N = 57 (N = 19 for all three sub-groups). Ns = not significant, BMI = body mass index, OAS = obstructive apnea syndrome, NRS = nutrition risk screening, ICU = intensive care unit, CRRT = continuous renal replacement therapy, SAPS II = Simplified Acute Physiology Score II, SOFA score = Sequential Organ Failure Assessment score. ^#^ P-value calculated using ANOVA or Krustkall-Wallis for continuous data and Fisher’s exact test for categorical data. ^a^ significantly different from “Respiratory cause of intubation” sub-group. ^b^ significantly different from “Neurological cause of intubation” sub-group. ^c^ significantly different from “Non-neurological & non-respiratory cause of intubation” sub-group.*
