# Supplementary material for: Early prediction of hospital outcomes in patients tracheostomized for complex mechanical ventilation weaning
Source: Ann Intensive Care. 2022 Aug 8;12:73. doi: 10.1186/s13613-022-01047-z (PMC9357593; doi:10.1186/s13613-022-01047-z)
Supplement: Supplementary file 2 — Additional file 2. Patients’ general characteristics, comorbidities and admission data for study population andseparated by cause for intubation. [file 13613_2022_1047_MOESM2_ESM.docx]

# Additional file 3

General characteristics & comorbidities, admission data, ventilation data, sedation, opioids, NMBA use, tracheostomy data and outcomes data for patients intubated for non-neurological reasons

|  | **Study population** | **Favourable outcome** | **Poor outcome** |  |
| --- | --- | --- | --- | --- |
|  | N = 57* | N = 39* | N = 18* | *p-value* |
| **General characteristics & comorbidities** | |  |  |  |
| Age - yr | 60 [52 - 73] | 59 [51 - 68] | 70.5 [58 - 78] | *0.02* |
| Women – n. (%) | 15 (26.3%) | 12 (30.8%) | 3 (16.7%) | *0.34* |
| BMI - kg/m^2^ | 24.5 [21 - 29] | 23.7 [21 - 28] | 27.3 [23 - 32] | *0.05* |
| Clinical Frailty Score | 4 [3 - 6] | 4 [3 - 6] | 4 [3 - 5] | *0.51* |
| NRS score at admission | 6 [3 - 6] | 6 [3 - 6] | 6 [3 - 7] | *0.47* |
| *Home O_2_-therapy – n. (%)* | 2 (3.5%) | 2 (5.1%) | 0 (0%) | *1* |
| *Home NIV-therapy – n. (%)* | 2 (3.5%) | 2 (5.1%) | 0 (0%) | *1* |
| **Admission data** |  |  |  |  |
| Type of ICU admission |  |  |  | *1* |
| *Medical – n. (%)* | 22 (38.6%) | 15 (38.5%) | 7 (38.9%) |  |
| *Surgical – n. (%)* | 35 (61.4%) | 24 (61.5%) | 11 (61.1%) |  |
| SAPS II at admission | 47 [39 - 65] | 45 [36 - 65] | 57 [43 - 68] | *0.22* |
| SOFA Score at admission | 9 [7 - 12] | 9 [8 - 12] | 9.5 [7 - 11] | *0.87* |
| **Ventilation data between intubation and tracheostomy** | |  |  |  |
| Percentage of mechanical ventilation days  with > 12 hours of: | |  |  |  |
| *VAC – n. (%)* | 42.9% [20 - 63%] | 43.8% [22 - 60%] | 37.3% [14 - 73%] | *0.84* |
| *PAC – n. (%)* | 0% [0 - 0%] | 0% [0 - 0%] | 0% [0 - 0%] | *0.54* |
| *PSV – n. (%)* | 54.5% [32 - 74%] | 54.6% [33 - 73%] | 53.7% [27 - 81%] | *0.93* |
| *Other – n. (%)* | 0% [0 - 0%] | 0% [0 - 0%] | 0% [0 - 0%] | *1* |
| V_T_ - mL | 451.6 [403 - 528] | 430.9 [401 - 510] | 469.5 [445 - 535] | *0.13* |
| V_T_/PBW - mL/kg | 6.8 [6 - 8] | 6.8 [6 - 8] | 6.8 [6 - 8] | *0.66* |
| PEEP - cmH_2_O | 7.5 [6 - 9] | 7.2 [6 - 9] | 7.5 [6 - 9] | *0.96* |
| RR - cycle/min | 22.5 [20 - 25] | 22.7 [20 - 25] | 21.5 [19 - 26] | *0.74* |
| Dynamic P_plat_ - cmH_2_O | 22.1 [20 - 25] | 22.5 [20 - 26] | 20.3 [20 - 23] | *0.15* |
| Driving pressure - cmH_2_O | 14.2 [12 – 17] | 14.6 [13 – 18] | 14.1 [12 – 15] | *0.17* |
| Separation attempts |  |  |  | *0.84* |
| *0* | 16 (28.1%) | 10 (25.6%) | 6 (33.3%) |  |
| *1* | 9 (15.8%) | 7 (17.9%) | 2 (11.1%) |  |
| *2* | 8 (14%) | 5 (12.8%) | 3 (316.7%) |  |
| *>2* | 24 (42.1%) | 17 (43.6%) | 7 (38.9%) |  |
| **Percentage of days with sedation use** | |  |  |  |
| Any sedation - % | 100% [90 - 100%] | 100% [92 - 100%] | 94.6% [86 - 100%] | *0.19* |
| Propofol - % | 78.6% [56 - 100%] | 80% [61 - 100%] | 72.9% [51 - 91%] | *0.47* |
| Midazolam - % | 33.3% [13 - 58%] | 36.4% [16 - 60%] | 15.5% [11 - 59%] | *0.24* |
| Dexmedetomidine - % | 11.8% [0 - 23%] | 16.7% [0 - 33%] | 8.1% [0 - 16%] | *0.49* |
| **Percentage of days with opioids use** | |  |  | |
| Opioids - % | 100% [91 - 100%] | 100% [91 - 100%] | 100% [92 - 100%] | *0.82* |
| Morphine - % | 0% [0 - 0%] | 0% [0 - 0%] | 0% [0 - 15%] | *0.12* |
| Fentanyl - % | 100% [87 - 100%] | 100% [89 - 100%] | 98.2% [63 - 100%] | *0.63* |
| Other opioids - % | 0% [0 - 0%] | 0% [0 - 0%] | 0% [0 - 1%] | *0.18* |
| **Percentage of days with NMBA use** | |  |  |  |
| NMBA - % | 16.7% [8 - 40%] | 16.7% [8 - 38%] | 15.5% [9 - 46%] | *0.70* |
| **Proportion of patients receiving sedation or opioids the day before tracheostomy** | |  |  |  |
| Sedation – n (%) | 49 (86%) | 35 (89.7%) | 14 (77.8%) | *0.25* |
| Opioids – n (%) | 51 (89.5%) | 36 (92.3%) | 15 (83.3%) | *0.37* |
| **Tracheostomy data** |  |  |  |  |
| Worst PaO_2_/FiO_2_ ratio on the day of tracheostomy |  |  |  | *0.92* |
| *≥ 400 mmHg* | 0 (0%) | 0 (0%) | 0 (0%) |  |
| *< 400 mmHg* | 5 (8.8%) | 3 (7.7%) | 2 (11.1%) |  |
| *< 300 mmHg* | 12 (21.1%) | 9 (23.1%) | 3 (16.7%) |  |
| *< 200 mmHg* | 36 (63.2%) | 24 (61.5%) | 12 (66.7%) |  |
| *< 100 mmHg* | 4 (7%) | 3 (7.7%) | 1 (5.6%) |  |
| Type of tracheostomy |  |  |  | *0.17* |
| *Percutaneous – n. (%)* | 5 (8.8%) | 5 (12.8%) | 0 (0%) |  |
| *Surgical – n. (%)* | 52 (91.2%) | 34 (87.2%) | 18 (100%) |  |
| Time from intubation to tracheostomy - days | 15.4 [11 - 21] | 13.3 [11 - 20] | 16.8 [14 - 25] | *0.14* |
| **General hospital data** |  |  |  |  |
| ICU stay duration - days | 39 [24 - 47] | 36 [21 - 47] | 42.5 [30 - 47] | *0.22* |
| Tertiary hospital stay duration - days | 57 [46 - 89] | 64 [49 - 99] | 52 [43 - 69] | *0.17* |
| Days free of MV at day 30 - days | 26 [13 - 39] | 27.7 [19 - 41] | 9.4 [0 - 31] | *0.01* |
| Days free of MV at day 60 - days | 0 [0 - 10] | 0 [0 - 11] | 0 [0 - 1] | *0.12* |
| Intubation to cannula ablation during or after acute care hospital stay - days | 45.5 [37 - 60] | 43 [37 - 56] | 93.5 [63 - 160] | *<0.01* |
| ICU-acquired weakness diagnosis – n. (%) | 19 (33.3%) | 13 (33.3%) | 6 (33.3%) | *1* |
| *with MRC score < 48/60 – n. (%)* | 16 (28.1%) | 11 (28.2%) | 5 (27.8%) | *1* |
| *with EMG / high clinical suspicion – n. (%)* | 3 (5.3%) | 2 (5.1%) | 1 (5.6%) | *1* |
| *MRC score value* | 21.5 [2.5 – 32.8] | 20 [0 – 33] | 23 [9.5 – 39] | *0.56* |

*N = 57 except for NRS where N = 38 (N = 26 for favourable and N = 12 for poor outcome), dynamic Pplat and driving pressure where N = 51 (N = 35 and N = 16), days free of MV at day 30 and at day 60 where N = 51 (N=36 and N = 15) and intubation to cannula ablation during or after acute care hospital stay where N = 42 (N = 38 and N = 4). BMI = body-mass index, NRS = nutrition risk screening, ICU VAC = volume assist-control, PSV = pressure-support ventilation, PAC = pressure assist-control ventilation, VT = tidal volume, PBW = predicted body-weight, PEEP = positive end-expiratory pressure, RR = respiratory rate, P_plat_ = plateau pressure, NMBA = neuromuscular blocking agents, EMNG = electromyography. V_T_, V_T_/PBW, PEEP, RR and Dynamic P_plat_ were recorded once a day at 8 am. # P-value calculated using T-test or Mann-Whitney test for continuous data and Fisher’s exact test for categorical data.*
