# Supplementary material for: Early prediction of hospital outcomes in patients tracheostomized for complex mechanical ventilation weaning
Source: Ann Intensive Care. 2022 Aug 8;12:73. doi: 10.1186/s13613-022-01047-z (PMC9357593; doi:10.1186/s13613-022-01047-z)
Supplement: Supplementary file 3 — Additional file 3. General characteristics & comorbidities, admission data, ventilation data, sedation, opioids, NMBA use, tracheostomy data and outcomes data for patients intubated for non-neurological reasons. [file 13613_2022_1047_MOESM3_ESM.docx]

# Additional file 4

General characteristics & comorbidities, admission data, ventilation data, sedation, opioids, NMBA use, tracheostomy data and outcomes data with patients intubated for neurological reasons only.

|  | **Study population** | **Favourable outcome** | **Poor outcome** |  |
| --- | --- | --- | --- | --- |
|  | N = 23* | N = 11* | N = 12* | *p-value* |
| **General characteristics & comorbidities** | |  |  |  |
| Age - yr | 59 [48 - 70] | 59 [39 - 66] | 64 [50 - 71] | 0.27 |
| Women – n. (%) | 8 (34.8%) | 4 (36.4%) | 4 (33.3%) | 1 |
| BMI - kg/m^2^ | 26.4 [24 - 31] | 24.8 [21 - 26] | 29.9 [26 - 33] | 0.01 |
| Clinical Frailty Score | 2 [2 - 3] | 2 [2 - 3] | 2 [2 - 4] | 0.88 |
| NRS score at admission | 4 [3 - 6] | 3 [3 - 5] | 4.5 [4 - 6] | 0.15 |
| *Home O_2_-therapy – n. (%)* | 0 (0%) | 0 (0%) | 0 (0%) | *1* |
| *Home NIV-therapy – n. (%)* | 1 (4.3%) | 0 (0%) | 1 (8.3%) | *1* |
| **Admission data** |  |  |  |  |
| Type of ICU admission |  |  |  | 1 |
| *Medical – n. (%)* | 6 (26.1%) | 3 (27.3%) | 3 (25%) |  |
| *Surgical – n. (%)* | 17 (73.9%) | 8 (72.7%) | 9 (75%) |  |
| SAPS II at admission | 46 [41 - 60] | 43 [31 - 63] | 48.5 [42 - 59] | 0.89 |
| SOFA Score at admission | 8 [6 - 10] | 8 [6 - 8] | 8.5 [6 - 10] | 0.42 |
| **Ventilation data between intubation and tracheostomy** |  |  |  |  |
| Percentage of mechanical ventilation days with > 12 hours of: |  |  |  |  |
| *VAC – n. (%)* | 25% [6 - 33%] | 26.7% [11 - 44%] | 18.3% [2 - 33%] | 0.26 |
| *PAC – n. (%)* | 0% [0 - 0%] | 0% [0 - 0%] | 0% [0 - 0%] | 1 |
| *PSV – n. (%)* | 72.2% [63 - 89%] | 66.7% [56 - 89%] | 80% [67 - 92%] | 0.21 |
| *Other – n. (%)* | 0% [0 - 0%] | 0% [0 - 0%] | 0% [0 - 0%] | 0.48 |
| V_T_ - mL | 476.8 [426 - 540] | 500.1 [430 - 540] | 470.4 [414 - 529] | 0.57 |
| V_T_/PBW - mL/kg | 7.7 [7 - 8] | 7.7 [7 - 8] | 7.7 [7 - 8] | 0.83 |
| PEEP - cmH_2_O | 5.3 [5 - 7] | 5.3 [5 - 7] | 5.9 [5 - 7] | 0.27 |
| RR - cycle/min | 21 [17 - 25] | 21 [19 - 25] | 21.2 [17 - 26] | 0.98 |
| Dynamic P_plat_ - cmH_2_O | 17.8 [16 - 19] | 17.9 [15 - 19] | 16.4 [15 - 19] | 0.98 |
| Driving pressure – cmH_2_O | 10.5 [9 – 13] | 11.8 [9 – 14] | 10.5 [9 – 11] | 0.44 |
| Separation attempts |  |  |  | 0.52 |
| *0* | 5 (21.7%) | 1 (9.1%) | 4 (33.3%) |  |
| *1* | 2 (8.7%) | 1 (9.1%) | 1 (8.3%) |  |
| *2* | 3 (13%) | 1 (9.1%) | 2 (16.7%) |  |
| *>2* | 13 (56.5%) | 8 (72.7%) | 5 (41.7%) |  |
| **Percentage of days with sedation use** | |  |  |  |
| Any sedation - % | 72.2% [47 - 89%] | 72.2% [53 - 80%] | 72.3% [15 - 91%] | 0.73 |
| Propofol - % | 72.2% [26 - 80%] | 72.2% [40 - 78%] | 60.4% [15 - 83%] | 0.77 |
| Midazolam - % | 0% [0 - 25%] | 10.5% [0 - 37%] | 0% [0 - 19%] | 0.24 |
| Dexmedetomidine - % | 0% [0 - 0%] | 0% [0 - 19%] | 0% [0 - 0%] | 0.16 |
| **Percentage of days with opioids use** | |  |  | |
| Opioids - % | 87% [67 - 100%] | 86.7% [67 - 100%] | 93.5% [64 - 100%] | 0.64 |
| Morphine - % | 0% [0 - 0%] | 0% [0 - 0%] | 0% [0 - 0%] | 0.73 |
| Fentanyl - % | 66.7% [26 - 100%] | 63% [13 - 84%] | 82.4% [34 - 100%] | 0.21 |
| Other opioids - % | 0% [0 - 11%] | 0% [0 - 47%] | 0% [0 - 0%] | 0.13 |
| **Percentage of days with NMBA use** | |  |  |  |
| NMBA - % | 0% [0 - 11%] | 5.3% [0 - 13%] | 0% [0 - 6%] | 0.27 |
| **Proportion of patients receiving sedation or opioids the day before tracheostomy** | |  |  |  |
| Sedation – n (%) | 11 (47.8%) | 3 (27.3%) | 8 (66.7%) | 0.10 |
| Opioids – n (%) | 16 (69.6%) | 7 (63.6%) | 9 (75%) | 0.67 |
| **Tracheostomy data** |  |  |  |  |
| Worst PaO_2_/FiO_2_ ratio on the day of tracheostomy |  |  |  | *0.66* |
| *≥ 400 mmHg* | 2 (8.7%) | 1 (9.1%) | 1 (8.3%) |  |
| *< 400 mmHg* | 7 (30.4%) | 2 (18.2%) | 5 (41.7%) |  |
| *< 300 mmHg* | 6 (26.1%) | 4 (36.4%) | 2 (16.7%) |  |
| *< 200 mmHg* | 7 (30.4%) | 4 (36.4%) | 3 (25%) |  |
| *< 100 mmHg* | 1 (4.3%) | 0 (0%) | 1 (8.3%) |  |
| Type of tracheostomy |  |  |  | 1 |
| *Percutaneous – n. (%)* | 8 (34.8%) | 4 (36.4%) | 4 (33.3%) |  |
| *Surgical – n. (%)* | 15 (65.2%) | 7 (63.6%) | 8 (66.7%) |  |
| Time from intubation to tracheostomy - days | 11 [10 - 19] | 16.8 [10 - 20] | 10.6 [10 - 16] | 0.19 |
| **General hospital data** |  |  |  |  |
| ICU stay duration - days | 20 [15 - 26] | 22 [15 - 32] | 18 [14 - 25] | 0.18 |
| Tertiary hospital stay duration - days | 46 [31 - 58] | 55 [43 - 58] | 41 [22 - 56] | 0.16 |
| Days free of MV at day 30 - days | 39.8 [34 - 48] | 39.8 [37 - 47] | 38.1 [32 - 50] | 0.60 |
| Days free of MV at day 60 - days | 11.9 [6 - 19] | 9.8 [7 - 17] | 13.5 [4 - 20] | 0.65 |
| Intubation to cannula ablation during or after acute care hospital stay - days | 34.5 [31 - 39] | 34 [31 - 37] | 62 [NA] | NA |
| ICU-acquired weakness diagnosis – n. (%) | 1 (1.8%) | 0 (0%) | 1 (5.6%) | 1 |
| *with MRC score < 48/60 – n. (%)* | 1 (1.8%) | 0 (0%) | 1 (5.6%) | 1 |
| *with EMNG / high clinical suspicion – n. (%)* | 0 (0%) | 0 (0%) | 0 (0%) | NA |
| *MRC score value* | 0 [NA] | NA | 0 [NA] | NA |

*N = 23 except for NRS where N = 19 (N = 9 for favourable and N = 10 for poor outcome), dynamic P_plat_ and driving pressure where N = 19 (N = 10 and N = 9), days free of MV at day 30 and at day 60 where N = 21 (N = 11 and N = 10) and intubation to cannula ablation during or after acute care hospital stay where N = 12 (N = 11 and N = 1). BMI = body-mass index, NRS = nutrition risk screening, ICU VAC = volume assist-control, PSV = pressure-support ventilation, PAC = pressure assist-control ventilation, V_T_ = tidal volume, PBW = predicted body-weight, PEEP = positive end-expiratory pressure, RR = respiratory rate, P_plat_ = plateau pressure, NMBA = neuromuscular blocking agents, EMNG = electromyoneurography. V_T_, V_T_/PBW, PEEP, RR and Dynamic P_plat_ were recorded once a day at 8 am. # P-value calculated using T-test or Mann-Whitney test for continuous data and Fisher’s exact test for categorical data.*
