# Supplementary material for: Early prediction of hospital outcomes in patients tracheostomized for complex mechanical ventilation weaning
Source: Ann Intensive Care. 2022 Aug 8;12:73. doi: 10.1186/s13613-022-01047-z (PMC9357593; doi:10.1186/s13613-022-01047-z)
Supplement: Supplementary file 4 — Additional file 4. General characteristics & comorbidities, admission data, ventilation data, sedation, opioids, NMBA use, tracheostomy data and outcomes data with patients intubated for neurological reasons only. [file 13613_2022_1047_MOESM4_ESM.docx]

# Additional file 5

*Ventilation and norepinephrine use 2-hours before tracheostomy.*

|  |  | **Study population** |  | **Favourable outcome** |  | **Poor outcome** |  |
| --- | --- | --- | --- | --- | --- | --- | --- |
|  | N | N = 80* | N | N = 50* | N | N = 30* | *p-value* |
| **Ventilation data 2-hours before tracheostomy** | | |  |  |  |  |  |
| Controlled ventilation mode | 80 | 10 (12.5%) | 50 | 8 (16%) | 30 | 2 (6.7%) | *0.31* |
|  |  |  |  |  |  |  |  |
| PEEP – cmH_2_O | 79 | 6.0 [5 - 8] | 49 | 6.0 [5 - 8] | 30 | 6.0 [5 - 8] | *0.93* |
| FiO_2_ - % | 80 | 30.4 [28 - 41] | 50 | 32.4 [27 - 41] | 30 | 30.0 [30 - 42] | *0.89* |
| RR – cycle/min | 80 | 20.6 [17 - 26] | 50 | 20.6 [17 - 27] | 30 | 20.6 [16 - 26] | *0.82* |
| Norepinephrine use | 80 | 50 (62.5%) | 50 | 31 (62%) | 30 | 19 (63.3%) | *1* |

*N=80 except for PEEP where N = 79 (N = 49 for favourable outcome and N = 30 for poor outcome). Ns = not significant, PEEP = positive end-expiratory pressure, FiO2 = inspired fraction of oxygen, RR = respiratory rate. # P-value calculated using T-test or Mann-Whitney test for continuous data and Fisher’s exact test for categorical data.*
