# Supplementary material for: Early prediction of hospital outcomes in patients tracheostomized for complex mechanical ventilation weaning
Source: Ann Intensive Care. 2022 Aug 8;12:73. doi: 10.1186/s13613-022-01047-z (PMC9357593; doi:10.1186/s13613-022-01047-z)
Supplement: Supplementary file 5 — Additional file 5. Ventilation and norepinephrine use 2-hours before tracheostomy. [file 13613_2022_1047_MOESM5_ESM.docx]

# Additional file 6

Univariate and multivariate logistic regression models for factors potentially associated with bad outcome.

|  | **Univariate model** | | **Multivariate model** | | |
| --- | --- | --- | --- | --- | --- |
|  | **OR (CI 95%)** | ***P-value*** | **OR (CI 95%)** | **VIF** | ***P-value*** |
| **BMI** | 1.181 (1.07 - 1.32) | 0.0009 | 1.209 (1.09 - 1.37) | 1.015 | 0.0008 |
| **Age** | 1.038 (1.01 - 1.08) | 0.0253 | 1.045 (1.01 to 1.1) | 1.055 | 0.0417 |
| Sex | 2.061 (0.73 - 6.42) | 0.2967 |  |  |  |
| Number of comorbidities | 1.248 (0.73 - 2.15) | 0.4471 |  |  |  |
| Clinical Frailty Score | 0.986 (0.76 - 1.27) | 0.8269 |  |  |  |
| NRS score at ICU admission | 1.129 (0.88 - 1.48) | 0.385 |  |  |  |
| SAPS II at ICU admission | 1.011 (0.99 - 1.04) | 0.3227 |  |  |  |
| SOFA score at ICU admission | 1.010 (0.87 - 1.17) | 0.8776 |  |  |  |
| Type of ICU admission (medical/surgical) | 0.560 (0.22 - 1.44) | 0.3203 |  |  |  |
| Neurological cause for intubation | 0.849 (0.30 - 2.30) | 0.8038 |  |  |  |
| V_T_/PBW | 1.160 (0.81 - 1.68) | 0.5643 |  |  |  |
| PEEP | 1.026 (0.79 - 1.34) | 0.996 |  |  |  |
| Dynamic plateau pressure | 0.898 (0.78 – 1.02) | 0.1097 |  |  |  |
| **Percentage of days with sedation use** | 0.213 (0.03 - 1.26) | 0.1011 | 0.227 (0.03 - 1.72) | 1.059 | 0.1634 |
| Percentage of days with opioids use | 0.293 (0.02 - 5.01) | 0.7923 |  |  |  |
| Percentage of days with NMBA use | 0.592 (0.07 - 4.19) | 0.401 |  |  |  |
| Control ventilation before tracheostomy | 0.383 (0.07 - 1.97) | 0.1597 |  |  |  |
| 1st separation attempt | 1.062 (0.95 - 1.19) | 0.2298 |  |  |  |
| Any separation attempt | 0.564 (0.20 - 1.57) | 0.3983 |  |  |  |
| Sedation use (day before tracheostomy) | 1.556 (0.54 - 4.91) | 0.551 |  |  |  |
| Opioids use (day before tracheostomy) | 3.949 (0.96 - 26.82) | 0.2531 |  |  |  |
| Tracheostomy technique (percutaneous vs surgical) | 1.427 (0.42 - 5.70) | 0.728 |  |  |  |
| **Time from intubation to tracheostomy** | 1.006 (0.95 - 1.07) | 0.921 | 1.023 (0.95 - 1.10) | 1.048 | 0.5444 |

BMI = body mass index, NRS = nutrition risk screening, ICU = intensive care unit, SAPS II = Simplified Acute Physiology Score II, SOFA score = Sequential Organ Failure Assessment score, V_T_/PBW = tidal volume divided by predicted body weight, PEEP = positive end-expiratory pressure, NMBA = neuromuscular blocking agents. Left p-values calculated using univariate logistic regression for each variable. Right p-values calculated with multiple logistic regression model, which included BMI, age and sedation use.
