# Supplementary material for: Early prediction of hospital outcomes in patients tracheostomized for complex mechanical ventilation weaning
Source: Ann Intensive Care. 2022 Aug 8;12:73. doi: 10.1186/s13613-022-01047-z (PMC9357593; doi:10.1186/s13613-022-01047-z)
Supplement: Supplementary file 6 — Additional file 6. Univariate and multivariate logistic regression models for factors potentially associated with poor outcome. [file 13613_2022_1047_MOESM6_ESM.docx]

# Additional file 7

Univariate analyses and multivariate logistic regression model for factors potentially associated with poor outcome for patients intubated for non-neurological reasons only.

|  | **Univariate regression** | | **Multivariate model** | | |
| --- | --- | --- | --- | --- | --- |
|  | **OR (CI 95%)** | ***P-value*** | **OR (CI 95%)** | **VIF** | ***P-value*** |
| **BMI** | 1.125 (1.01 - 1.27) | 0.0503 | 1.151 (1.03 - 1.31) | 1.007 | 0.0228 |
| **Age** | 1.054 (1.01 - 1.11) | 0.0191 | 1.067 (1.01 - 1.14) | 1.007 | 0.0234 |
| Sex | 2.222 (0.59 - 10.88) | 0.3954 |  |  |  |
| Number of comorbidities | 0.993 (0.49 - 2.06) | 0.8976 |  |  |  |
| Clinical Frailty Score | 1.076 (0.80 - 1.46) | 0.5142 |  |  |  |
| NRS score at ICU admission | 1.132 (0.84 - 1.57) | 0.4798 |  |  |  |
| SAPS II at ICU admission | 1.018 (0.99 - 1.05) | 0.2133 |  |  |  |
| SOFA score at ICU admission | 1.035 (0.87 - 1.23) | 0.8637 |  |  |  |
| Type of ICU admission (medical/surgical) | 0.982 (0.31 - 3.19) | 0.9795 |  |  |  |
| V_T_/PBW | 1.061 (0.65 - 1.72) | 0.6553 |  |  |  |
| PEEP | 1.042 (0.74 - 1.47) | 0.9521 |  |  |  |
| Dynamic plateau pressure | 0.887 (0.73 – 1.05) | 0.1467 |  |  |  |
| Percentage of days with sedation use | 0.365 (0.02 - 8.12) | 0.2362 |  |  |  |
| Percentage of days with opioids use | 0.209 (0.00 - 66.93) | 0.8368 |  |  |  |
| Percentage of days with NMBA use | 1.817 (0.18 - 17.49) | 0.693 |  |  |  |
| Control ventilation before tracheostomy | 3.719 (0.59 - 72.48) | 0.4552 |  |  |  |
| 1st separation attempt | 1.069 (0.94 - 1.23) | 0.2532 |  |  |  |
| Any separation attempt | 0.690 (0.21 - 2.41) | 0.643 |  |  |  |
| Sedation use (day before tracheostomy) | 0.400 (0.08 - 1.90) | 0.4709 |  |  |  |
| Opioids use (day before tracheostomy) | 0.417 (0.07 - 2.47) | 0.5887 |  |  |  |
| Time from intubation to tracheostomy | 1.049 (0.98 - 1.13) | 0.1353 |  |  |  |

*BMI = body mass index, NRS = nutrition risk screening, ICU = intensive care unit, SAPS II = Simplified Acute Physiology Score II, SOFA score = Sequential Organ Failure Assessment score, V_T_/PBW = tidal volume divided by predicted body weight, PEEP = positive end-expiratory pressure, NMBA = neuromuscular blocking agents. Left p-values calculated using univariate logistic regression for each variable. Right p-values calculated with multiple logistic regression model, which included BMI and age.*
