# Supplementary material for: Early prediction of hospital outcomes in patients tracheostomized for complex mechanical ventilation weaning
Source: Ann Intensive Care. 2022 Aug 8;12:73. doi: 10.1186/s13613-022-01047-z (PMC9357593; doi:10.1186/s13613-022-01047-z)
Supplement: Supplementary file 7 — Additional file 7. Univariate analyses and multivariate logistic regression model for factors potentially associated with poor outcome for patients intubated for non-neurological reasons only. [file 13613_2022_1047_MOESM7_ESM.docx]

# Additional file 1

**General data and patients features:** general and demographic data were collected: age at time of hospital admission, sex, weight, height. Predicted body weight (PBW) was calculated using the ARDSnet method (See 2.2.8). Data concerning patient’s comorbidities at admission were also collected: chronic obstructive pulmonary disease (COPD), restrictive pulmonary disease, obstructive sleep apnea syndrome or other pulmonary disease. Coronary heart disease, heart failure, chronic kidney disease on dialysis or not, active neoplasia (neoplasia considered in remission were not counted) and peripheral or central neurological disease.

Data at ICU admission were collected as well: SAPS II (Simplified Acute Physiology Score II) and sequential organ failure assessment (SOFA) score were collected as well. Dates of admission and discharge from the hospital, date and hour of admission and discharge from the ICU were collected.

**Pre-tracheostomy data:** from the day after intubation to the day before tracheostomy, different data were collected. MV data included main mode of ventilation used (volume assist-control – VAC, pressure assist-control – PAC, pressure support ventilation – PSV and other modes). It also included tidal volume (V_T_), PEEP, respiratory rate, plateau pressure (P_plat_) and inspiratory pressure. Inspiratory pressure was defined as the maximal pressure during inspiration set on ventilator if the patient was ventilated on barometric mode (either assist-control pressure ventilation or pressure-support ventilation or other). Those data were collected at one consistent moment of the day (8 a.m.) as well as the lowest and highest value for each day. Maximum FiO_2_ was observed. Sedation, opioids and neuromuscular blocking agents (NMBA) use (bolus or continuous) during the day were collected as well. Sedation drugs included propofol, midazolam, dexmedetomidine and other sedation. Opioids use included fentanyl, morphine and other opioids (including sufentanil).

Spontaneous breathing trials (SBT) were gathered by analyzing ad-hoc pressure support (PS) and PEEP evolution day-by-day with minimal and maximal values. An SBT was defined as a day where pressure support was dropped to a minimal value of 8 cmH_2_O along with a minimal PEEP of 5 cmH_2_O. If, for subsequent days, PS remained the same value, those days were not counted as having a SBT. If PEEP was lowered to a minimal value of 0 cmH_2_O during a day along with a PS of 5-7 cmH_2_O, an SBT was counted.

Decannulation data was obtained, when missing, from institutions where patients stayed after the Lausanne University Hospital.

**Tracheostomy data**: the following data were collected on tracheostomy day: date and hour of tracheostomy and type of tracheostomy (surgical or percutaneous). Respiratory rate, FiO_2_ and PEEP were recorded every 30 minutes during the two hours prior to tracheostomy. MV mode used and norepinephrine use (maximum value) 2 hours prior to tracheostomy were also collected.

**Outcome data:** mortality (withdrawal of life-sustaining treatments or not) in the ICU or hospital stay (from discharge letters and clinical information system) was noted. ICU-acquired neuropathy and date of tracheostomy cannula ablation (decannulation) were also collected.

**Calculated data:** from collected data, following calculations were made:

1. Time from intubation to tracheostomy : tracheostomy day and hour - intubation day & hour

Time from intubation to decannulation : intubation day - decannulation day

1. Hospital stay : Lausanne hospital discharge day – Lausanne hospital admission day
2. ICU stay : Lausanne ICU discharge day – Lausanne ICU admission day
3. Body mass index (BMI) : admission weight (kg) / height (m)^2^
4. Predicted body weight (ARDSNET (ref)) : ♂: PBW (kg) = 50 + 2.3 (height (in) – 60) ┃ ♀: PBW (kg) = 45.5 + 2.3 (height (in) – 60)
